# 

**Published:** 1938

**Authors:** 


### CONTENTS.

| | Page |
|---|---|
| **The Twenty-seventh Long Fox Memorial Lecture: Air as a Conductor of Electricity.** By Professor A. M. TYNDALL, D.Sc., F.R.S. | 211 |
| **Syphilis in the Tropics.** By T. F. HEWER, M.D., M.R.C.P. | 217 |
| **A Guide Dog for the Blind.** By F. WILFRED WILLWAY, F.R.C.S. | 235 |
| **Drinker's "Iron Lung" and other Artificial Respirators.** By J. A. NIXON, C.M.G., M.D., F.R.C.P. | 239 |
| **Reviews of Books** | 245 |
| **Editorial Notes** | 260 |
| **Obituaries:** PATRICK WATSON-WILLIAMS, M.D. | 262 |
| HARRY FRANCOIS DEVIS, M.R.C.S., L.R.C.P. | 266 |
| NEIL PATRICK ESKELL, M.A. Cantab, M.R.C.S., L.R.C.P. | 266 |
| **Meetings of Societies** | 267 |
| **The Medical Library of the University of Bristol** | 270 |
| **Publications Received** | 272 |
| **Local Medical Notes** | 273 |

